# Prognostic Impact of Time to Castration Resistance on Overall Survival in Patients With Metastatic Castration‐Sensitive Prostate Cancer

**DOI:** 10.1111/iju.70197

**Published:** 2025-08-19

**Authors:** Hiroaki Iwamoto, Tomohiro Hori, Takahiro Inaba, Ryunosuke Nakagawa, Taiki Kamijima, Hiroshi Kano, Tomoyuki Makino, Renato Naito, Hiroshi Yaegashi, Takahiro Nohara, Kazuyoshi Shigehara, Kouji Izumi, Atsushi Mizokami

**Affiliations:** ^1^ Department of Integrative Cancer Therapy and Urology Kanazawa University Graduate School of Medical Science Kanazawa Japan

**Keywords:** androgen deprivation therapy, combined androgen blockade, metastatic castration‐sensitive prostate cancer, novel androgen receptor signaling inhibitor, overall survival, time to castration resistance

## Abstract

**Objectives:**

Treatment strategies for metastatic castration‐sensitive prostate cancer (mCSPC) have advanced significantly, yet the prognostic impact of time to castration resistance (TTCR) on overall survival (OS) remains unclear. This retrospective study aimed to evaluate the relationship between TTCR and OS.

**Methods:**

Among 218 patients diagnosed with pure prostatic adenocarcinoma and treated with combined androgen blockade therapy at Kanazawa University Hospital between 2000 and 2020, 160 who progressed to metastatic castration‐resistant prostate cancer (mCRPC) were included. OS was evaluated from initial diagnosis (OS‐PC) and from mCRPC progression (OS‐CRPC). TTCR was stratified into four groups. Kaplan–Meier analysis and log‐rank tests were used to assess survival. Outcomes were further analyzed based on the use of androgen receptor signaling inhibitors (ARSIs) post‐mCRPC progression.

**Results:**

The median follow‐up was 50.1 months. Median OS‐PC and OS‐CRPC were 70.6 and 48.1 months, respectively. Shorter TTCR and Gleason pattern 5 were associated with worse OS‐PC. ALP (IFCC) > 130 IU/L, LDH (IFCC) > 300 IU/L, and shorter TTCR predicted poorer OS‐CRPC. Among patients with TTCR ≥ 12 months, ARSI use post‐mCRPC progression was significantly associated with improved OS‐PC and OS‐CRPC. In contrast, for patients with TTCR < 12 months, ARSI treatment improved OS‐CRPC but not OS‐PC.

**Conclusions:**

Short TTCR was linked to reduced OS‐PC and OS‐CRPC. ARSI therapy after mCRPC progression provided substantial survival benefits, particularly in patients with TTCR ≥ 12 months. These findings may help reconsider the role of CAB in Asians, including Japanese.

## 1 Introduction

Prostate cancer (PC) is the most commonly diagnosed cancer in men and the leading cause of cancer‐related deaths in developed countries [1]. Approximately 10% of patients with distant metastases upon diagnosis are classified as having metastatic castration‐sensitive prostate cancer (mCSPC) [2]. Since the introduction of androgen deprivation therapy (ADT) in 1941, which targets androgen receptor (AR) signaling, ADT has remained the standard treatment for metastatic PC [3]. Combined androgen blockade (CAB), which combines a nonsteroidal anti‐androgen with either an LHRH agonist or bilateral orchiectomy, was developed to improve treatment outcomes. In recent years, novel androgen receptor signaling inhibitors (ARSIs) have become the mainstream treatment for mCSPC. Current guidelines recommend ADT combined with ARSIs (e.g., abiraterone, enzalutamide, or apalutamide) or triplet therapy involving ADT, docetaxel (DTX), and an ARSI (e.g., abiraterone or darolutamide) for patients with mCSPC [4]. In recent years, it has been indicated that prolonging the time to castration resistance (TTCR) through intensive treatment during the mCSPC stage may contribute to improved overall survival (OS). In fact, OS following progression to castration‐resistant prostate cancer (CRPC) has been reported to remain stable regardless of TTCR [5]. Notably, OS improves with the administration of a novel ARSI after mCRPC progression [6, 7]. Despite these insights, data on the prognostic impact of TTCR on OS remain limited. Therefore, we conducted a retrospective study to assess this relationship and also investigated the effect of ARSI administration after CRPC on OS.

## 2 Materials and Methods

### 2.1 Patient Selection

Among patients with mCSPC treated at Kanazawa University Hospital between January 2000 and December 2020, a total of 218 were diagnosed with pure adenocarcinoma and received CAB. Of these, 160 patients who progressed to mCRPC were included in this retrospective study.

### 2.2 Collection of Clinical Data

Demographic, pathological, and follow‐up data—including clinical stage, treatment details, lab results, CRPC progression date, and survival—were collected from medical records. Clinical staging followed the 2017 TNM classification (8th edition) [8]. PSA failure after ADT was defined as a ≥ 25% rise from the PSA nadir, confirmed ≥ 4 weeks later, with an absolute increase of ≥ 2.0 ng/mL. CRPC was diagnosed based on these criteria. Treatment regimens, testing schedules, and imaging intervals after mCSPC diagnosis were determined by each attending physician. The study follow‐up ended on December 31, 2023, with a median duration of 50.1 months.

### 2.3 Statistical Analyses

OS and TTCR were estimated using the Kaplan–Meier method, with survival differences assessed by the log‐rank test. OS was evaluated from PC diagnosis (OS‐PC) and CRPC progression (OS‐CRPC). TTCR was stratified into four categories: ≥ 18 months, 12 < TTCR < 18 months, 6 < TTCR < 12 months, and < 6 months. To evaluate trends across categories, TTCR was modeled as an ordinal variable. Hazard ratios (HRs) and 95% confidence intervals (CIs) were calculated, and multivariate analysis was performed using the Cox proportional hazards model. Statistical analyses were conducted using SPSS (v25.0) and GraphPad Prism (v9). A *p* value < 0.05 was considered statistically significant.

### 2.4 Ethical Considerations

This study was approved by the Institutional Review Board of Kanazawa University Hospital (Approval No. 2016–328). Informed consent was obtained via an opt‐out process approved by the Medical Ethics Committee. All procedures adhered to relevant ethical guidelines and regulations.

## 3 Results

### 3.1 Patient Characteristics

Table 1 summarizes the characteristics of the 160 mCSPC patients included. The median age at diagnosis was 70 years. Median baseline levels were: PSA 244 ng/mL, hemoglobin (Hb) 13.3 g/dL, lactate dehydrogenase (LDH, IFCC) 192.5 IU/L, and alkaline phosphatase (ALP, IFCC) 124.5 IU/L. All patients received CAB. Grade Group ≥ 4 was observed in 138 patients (86.3%), and 18 (11.3%) had M1c disease. After CRPC progression, 93 (58.1%) received ARSIs, and 80 (50.0%) underwent chemotherapy. Patients were stratified by TTCR: ≥ 18 months (22; 13.8%), 12–18 months (58; 36.3%), 6–12 months (32; 20.0%), and < 6 months (48; 30.0%). Shorter TTCR was associated with higher ALP and higher Grade Group. Table S1 shows that shorter TTCR correlated with elevated PSA, Hb, and ALP at mCRPC progression. Table S2 presents the breakdown of visceral metastases at the time of prostate cancer diagnosis and at mCRPC progression.

**TABLE 1:** Patient characteristics.

Characteristics	All	Time to castration resistance, months				*p*
		≤ 6	6 < TTCR ≤ 12	12 < TTCR ≤ 18	> 18	
Patients, *n*	160	22 (13.8)	58 (36.3)	32 (20.0)	48 (30.0)	
Median age, years	70	71.5	72.5	69	71	0.61
Median PSA, ng/mL	244	230.8	309.5	352	218.3	0.3
Median Hb, g/dL	13.3	13.0	13.0	13.2	13.6	0.55
Median LDH (IFCC), IU/L	192.5	209.5	194.5	191	178	0.36
Median ALP (IFCC), IU/L	124.5	174.5	161	105	91	0.032
Histology, *n* (%)						0.006
Grade group ≤ 3	18 (11.3)	0	4 (6.9)	6 (18.8)	8 (16.7)	
Grade group = 4	42 (26.3)	3 (13.6)	12 (20.7)	13 (40.6)	14 (29.2)	
Grade group = 5	96 (60.0)	19 (86.4)	42 (72.4)	12 (37.5)	23 (47.9)	
Unknown	4 (2.5)	0	0	1 (3.1)	3 (6.3)	
*N* stage, *n* (%)						0.2
N0	61 (38.1)	9 (40.9)	20 (34.5)	17 (53.1)	15 (31.3)	
N1	97 (60.6)	13 (59.1)	38 (65.5)	15 (46.9)	31 (64.6)	
Visceral metastasis, *n* (%)						0.46
No	142 (88.8)	21 (95.5)	49 (84.5)	28 (87.5)	44 (91.7)	
Yes	18 (11.3)	1 (4.5)	9 (15.5)	4 (12.5)	4 (8.3)	
Lattitude						0.0005
Low	46 (28.8)	2 (9.1)	10 (17.2)	11 (34.4)	23 (47.9)	
High	109 (68.1)	20 (90.9)	46 (79.3)	20 (62.5)	23 (47.9)	
CHAARTED						0.0002
Low	49 (30.6)	2 (9.1)	10 (17.2)	15 (46.9)	22 (45.8)	
High	106 (66.3)	20 (90.9)	46 (79.3)	16 (50.0)	24 (50.0)	
All‐cause death, *n* (%)	92 (57.5)	18 (81.8)	35 (60.3)	20 (62.5)	19 (39.6)	0.01
PC‐specific mortality, *n* (%)	79 (49.4)	18 (81.8)	31 (53.4)	17 (53.1)	13 (27.1)	0.001

Abbreviations: ALP, alkaline phosphatase; CRPC, castration‐resistant prostate cancer; Hb, hemoglobin; LDH, lactate dehydrogenase; PC, prostate cancer; PSA, prostate‐specific antigen; TTCR: time to castration resistance.

### 3.2 Identification of Prognostic Factors in OS From PC Diagnosis

Multivariate Cox analysis was conducted to identify predictors of OS‐PC (Table 2), treating TTCR as an ordinal variable in four categories: ≥ 18 months, 12–18 months, 6–12 months, and < 6 months. GP5 (HR 2.22; 95% CI, 1.16–4.27; *p* = 0.017) and shorter TTCR (HR 2.60; 95% CI, 1.70–3.30; *p* < 0.001) were significant predictors of worse OS‐PC.

**TABLE 2:** Prognostic factors predicting OS after PC diagnosis.

		*n*	Univariate		Multivariate	
			HR (95% CI)	*p*	HR (95% CI)	*p*
Age at diagnosis, years	≥ 70	89	1.67 (1.06–2.63)	0.03	1.72 (0.98–3.02)	0.06
	< 70 (Ref.)	71				
PSA, ng/mL	≥ 100	111	0.89 (0.57–1.39)	0.61		
	< 100 (Ref.)	48				
Hb, g/dL	< 12	28	0.98 (0.53–1.79)	0.94		
	≥ 12 (Ref.)	80				
ALP (IFCC), IU/L	≥ 130	54	1.39 (0.83–2.33)	0.21		
	< 130 (Ref.)	56				
LDH (IFCC), IU/L	≥ 300	8	3.47 (1.45–8.30)	0.005	2.31 (0.96–5.58)	0.06
	< 300 (Ref.)	101				
Gleason pattern 5	Yes	97	2.66 (1.67–4.23)	< 0.001	2.22 (1.16–4.27)	0.017
	No (Ref.)	60				
Visceral metastasis	Yes	18	0.85 (0.40–1.65)	0.63		
	No (Ref.)	142				
TTCR, months	TTCR > 18	49	2.60 (2.03–3.34)	< 0.001	2.36 (1.70–3.30)	< 0.001
(ordinal)	12 < TTCR ≤ 18	31				
	6 < TTCR ≤ 12	58				
	TTCR ≤ 6	22				

Abbreviations: ALP, alkaline phosphatase; CI, confidence interval; Hb, hemoglobin; HR, hazard ratio; LDH, lactate dehydrogenase; OS, overall survival; PC, prostate cancer; PSA, prostate‐specific antigen; TTCR, time to castration resistance.

### 3.3 Kaplan–Meier Curves of OS‐PC

**FIGURE 1:**
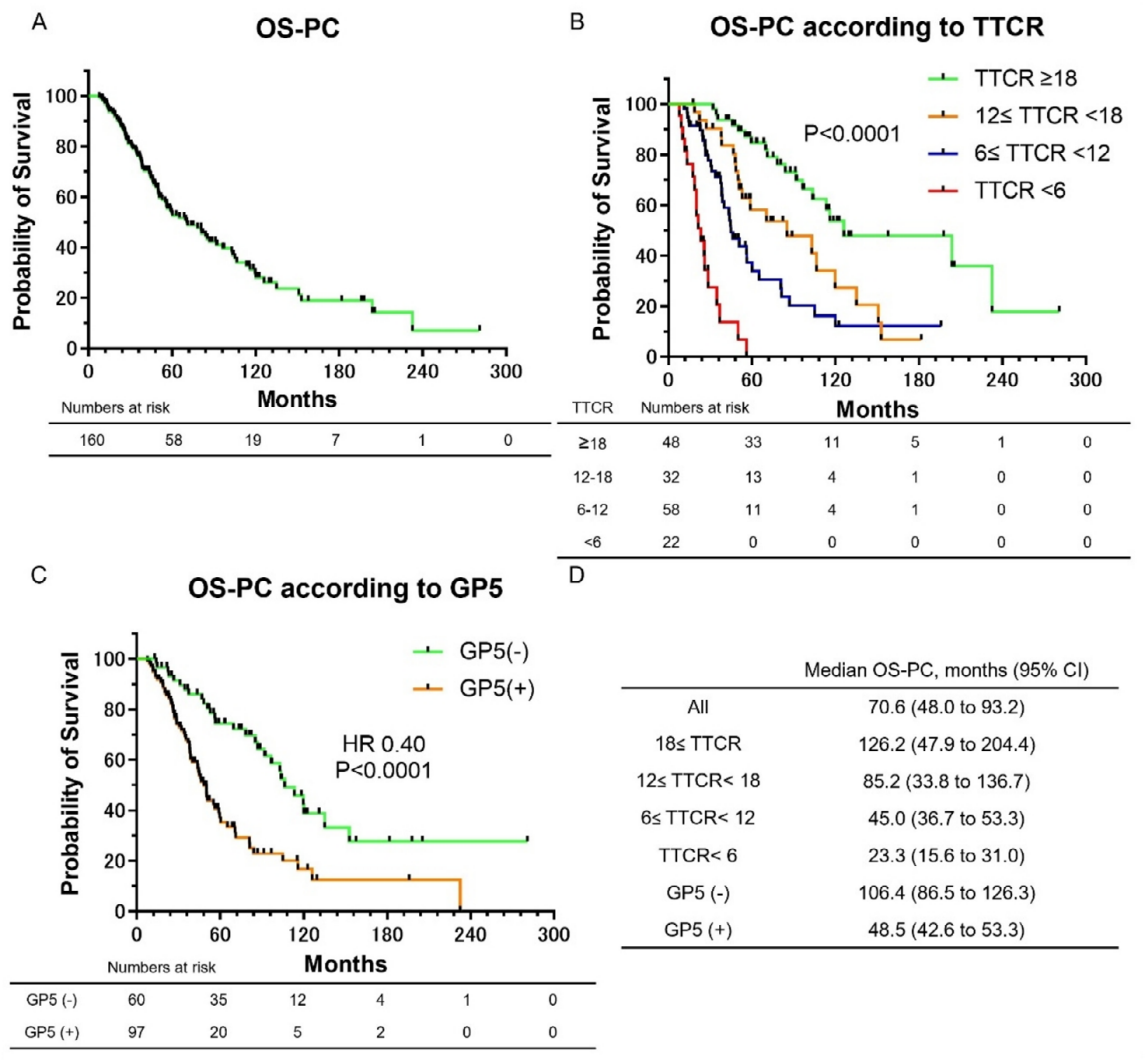
Kaplan–Meier curves illustrating OS‐PC. (A) Kaplan–Meier curves of OS‐PC in all patients. (B) Kaplan–Meier curves of OS‐PC according to TTCR. (C) Kaplan–Meier curves of OS‐PC according to GP5. (D) Median OS‐PC for each group.

Kaplan–Meier survival curves for OS‐PC are shown in Figure 1A–C. As shown in Figure 1A, the median OS‐PC for all patients was 70.6 months. Figure 1B shows OS‐PC stratified by TTCR. The median OS‐PC was 126.2 months in the TTCR ≥ 18 month group, 85.2 months in the 12 ≤ TTCR < 18 month group, 45.0 months in the 6 ≤ TTCR < 12 month group, and 23.3 months in the TTCR < 6 month group (*p* < 0.0001). A shorter TTCR was significantly associated with a reduced OS‐PC. As shown in Figure S1, even when limited to the high‐volume group defined by the CHAARTED criteria, a shorter TTCR was significantly associated with a reduced OS‐PC. Figure 1C depicts OS‐PC stratified by the presence of GP5. The median OS‐PC was 106.4 months in the GP5‐negative group and 48.5 months in the GP5‐positive group, with the latter showing significantly worse survival (HR: 0.40, *p* < 0.0001). Figure 1D shows the median OS‐PC for each group.

### 3.4 Identification of Prognostic Factors in OS From CRPC Progression

Multivariate analysis was performed using the Cox proportional hazards model to examine factors predicting OS‐CRPC (Table 3). TTCR was stratified into four categories—TTCR ≥ 18 months, 12 ≤ TTCR < 18 months, 6 ≤ TTCR < 12 months, and TTCR < 6 months—and treated as an ordinal variable. ALP (IFCC) > 130 IU/L (HR: 3.65, 95% CI: 1.85–7.18, *p* < 0.001), LDH (IFCC) > 300 IU/L (HR: 2.21, 95% CI: 1.10–4.45, *p* = 0.03), and shorter TTCR (HR: 1.54, 95% CI: 1.12–2.11, *p* = 0.008) were independent predictors of worse OS‐CRPC.

**TABLE 3:** Prognostic factors predicting OS after CRPC progression.

		*n*	Univariate		Multivariate	
			HR (95% CI)	*p*	HR (95% CI)	*p*
Age at diagnosis, years	≥ 70	96	1.51 (0.98–2.33)	0.07		
	< 70 (Ref.)	64				
PSA, ng/mL	≥ 100	59	2.45 (1.55–3.85)	< 0.001	0.87 (0.44–1.69)	0.68
	< 100 (Ref.)	87				
Hb, g/dL			1.64 (0.97–2.78)	0.07		
	≥ 12 (Ref.)	77				
ALP (IFCC), IU/L	< 12	36	4.82 (2.79–8.32)	< 0.001	3.65 (1.85–7.18)	< 0.001
	< 130 (Ref.)	80				
LDH (IFCC), IU/L	≥ 130	32	4.17 (2.25–7.75)	< 0.001	2.21 (1.10–4.45)	0.03
	< 300 (Ref.)	96				
Gleason pattern 5	Yes	97	2.62 (1.64–4.19)	< 0.001	1.53 (0.79–2.93)	0.21
	No (Ref.)	60				
Visceral metastasis	Yes	23	2.41 (1.37–4.22)	0.002	1.63 (0.89–2.99)	0.12
	No (Ref.)	107				
TTCR, months	TTCR > 18	49	2.03 (1.60–2.57)	< 0.001	1.54 (1.12–2.11)	0.008
(ordinal)	12 < TTCR ≤ 18	31				
	6 < TTCR ≤ 12	58				
	TTCR ≤ 6	22				

Abbreviations: ALP, alkaline phosphatase; CI, confidence interval; CRPC, castration‐resistant prostate cancer; Hb, hemoglobin; HR, hazard ratio; LDH, lactate dehydrogenase; OS, overall survival; PSA, prostate‐specific antigen; TTCR, time to castration resistance.

### 3.5 Kaplan–Meier Curves of OS‐CRPC

**FIGURE 2:**
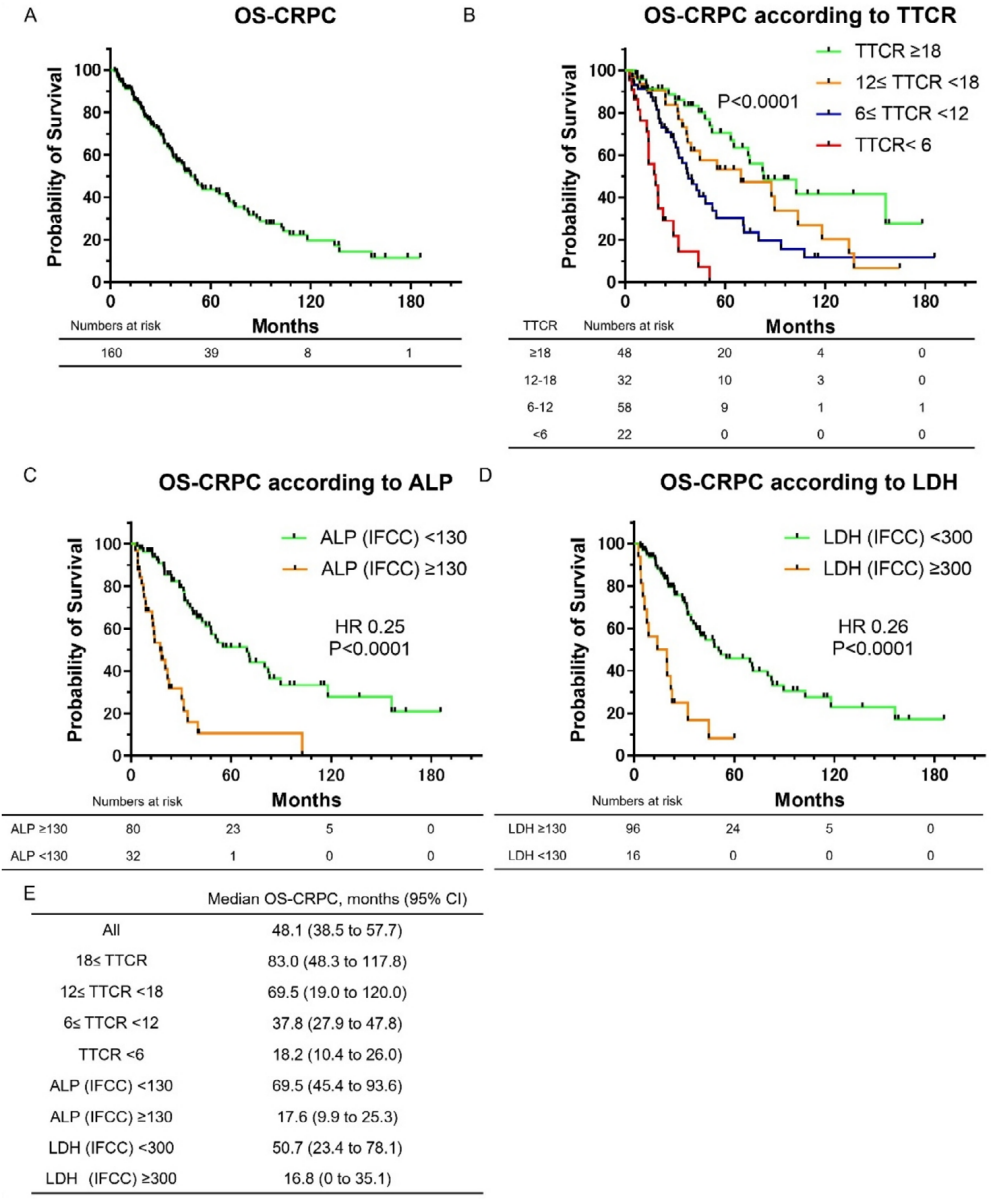
Kaplan–Meier curves showing OS‐CRPC. (A) Kaplan–Meier curves for all patients. (B) Kaplan–Meier curves of OS‐CRPC by TTCR. (C) Kaplan–Meier curves of OS‐CRPC stratified by ALP. (D) Kaplan–Meier curves of OS‐CRPC stratified by LDH levels. (E) Median OS‐CRPC for each group.

Kaplan–Meier survival curves for OS‐CRPC are shown in Figure 2A–C. The median OS‐CRPC for all patients was 48.1 months (Figure 2A). Figure 2B presents OS‐CRPC stratified by TTCR. The median OS‐CRPC was 83.0 months in the TTCR ≥ 18 month group, 69.5 months in the 12 ≤ TTCR < 18 month group, 37.8 months in the 6 ≤ TTCR < 12 month group, and 18.2 months in the TTCR < 6 month group (*p* < 0.0001). A shorter TTCR was significantly associated with shorter OS‐CRPC. As shown in Figure S1, even when limited to the high‐volume group defined by the CHAARTED criteria, a shorter TTCR was significantly associated with a reduced OS‐CRPC. Figure 2C shows OS‐CRPC stratified by ALP (IFCC) levels. The median OS‐CRPC was 69.5 months in patients with ALP (IFCC) < 130 IU/L and 17.6 months in those with ALP (IFCC) ≥ 130 IU/L, with significantly shorter survival in the latter group (HR: 0.25, *p* < 0.0001). Figure 2D presents OS‐CRPC according to LDH (IFCC) levels. The median OS‐CRPC was 50.7 months in the LDH (IFCC) < 300 IU/L group and 16.8 months in the LDH (IFCC) ≥ 300 IU/L group, again showing significantly poorer survival in the elevated LDH group (HR: 0.26, *p* < 0.0001). Figure 2D shows the median OS‐CRPC for each group.

### 3.6 Kaplan–Meier Curves of OS‐PC Stratified by the Administration of ARSIs as a Subsequent Treatment

**FIGURE 3:**
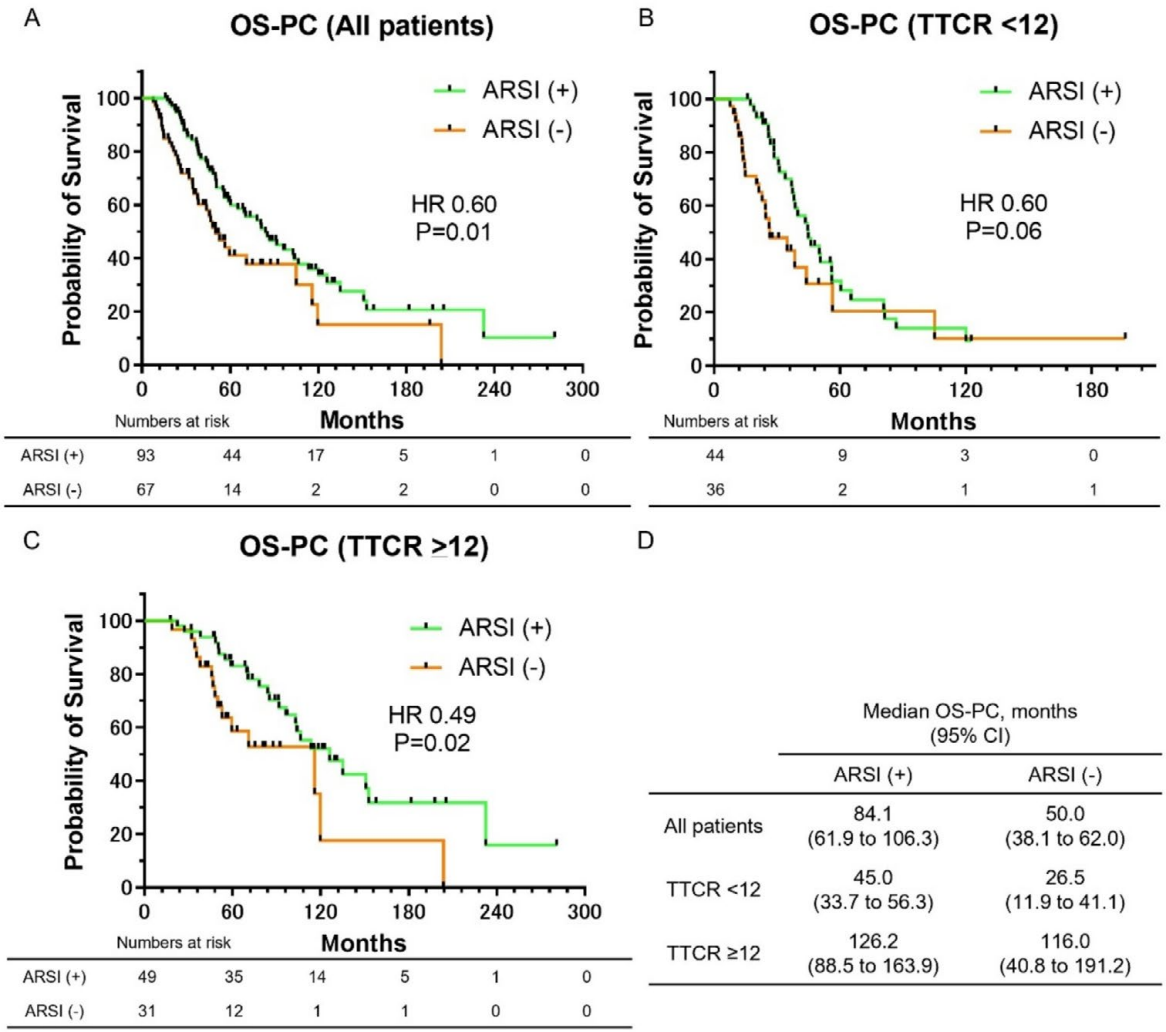
Kaplan–Meier survival curves for OS‐PC, stratified by the administration of ARSIs as a subsequent treatment. (A) OS‐PC in all patients, stratified by ARSI administration. (B) OS‐PC in patients with TTCR < 12 months, stratified by ARSI administration. (C) OS‐PC in patients with TTCR ≥ 12 months, stratified by ARSI administration. (D) Median OS‐PC for each subgroup.

Kaplan–Meier survival curves for OS‐PC, stratified by the administration of ARSIs as a subsequent treatment, are shown in Figure 3A–C. As shown in Figure 3A, the median OS‐PC was 84.1 months in the ARSI (+) group and 50.0 months in the ARSI (−) group, with significantly longer survival in the ARSI (+) group (HR: 0.60, *p* = 0.01). For subgroup analyses, patients were stratified according to TTCR. In patients with TTCR < 12 months (Figure 3B), the median OS‐PC was 45.0 months in the ARSI (+) group and 26.5 months in the ARSI (−) group. Although the ARSI (+) group had longer survival, the difference was not statistically significant (HR: 0.60, *p* = 0.06). In patients with TTCR ≥ 12 months (Figure 3C), the median OS‐PC was 126.2 months in the ARSI (+) group and 116.0 months in the ARSI (−) group, showing significantly longer survival in the ARSI (+) group (HR: 0.49, *p* = 0.02). The median OS‐PC for each group is presented in Figure 3D.

### 3.7 Kaplan–Meier Curves of OS‐CRPC Stratified by the Administration of ARSIs as a Subsequent Treatment

**FIGURE 4:**
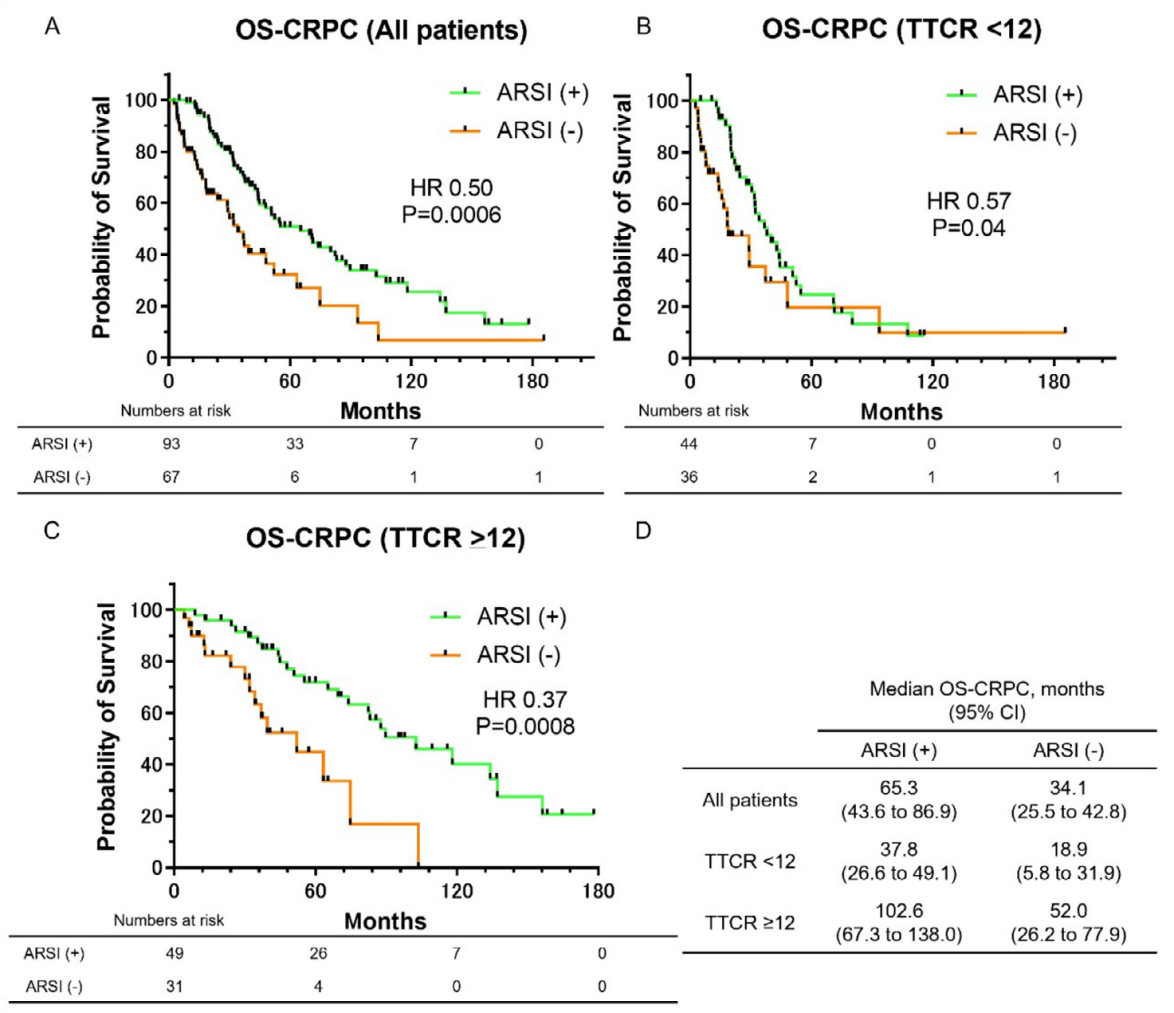
Kaplan–Meier survival curves for OS‐CRPC, stratified by administration of ARSIs as a subsequent treatment. (A) OS‐CRPC in all patients. (B) OS‐CRPC in patients with TTCR < 12 months. (C) OS‐CRPC in patients with TTCR ≥ 12 months. (D) Median OS‐CRPC for each group.

Kaplan–Meier survival curves for OS‐CRPC, stratified by ARSI administration of ARSIs as a subsequent treatment, are shown in Figure 4A–C. As shown in Figure 4A, the median OS‐CRPC was significantly longer in the ARSI (+) group (65.3 months) compared to the ARSI (−) group (34.1 months) (HR: 0.50, *p* = 0.0006). To further assess the impact of TTCR on treatment outcomes, patients were stratified by TTCR. In patients with TTCR < 12 months (Figure 4B), the median OS‐CRPC was 37.8 months in the ARSI (+) group and 18.9 months in the ARSI (−) group, showing significantly longer survival in the ARSI (+) group (HR: 0.57, *p* = 0.04). Among patients with TTCR ≥ 12 months (Figure 4C), the median OS‐CRPC was 102.6 months in the ARSI (+) group and 52.0 months in the ARSI (−) group, again showing significantly longer survival in the ARSI (+) group (HR: 0.37, *p* = 0.0008). The median OS‐CRPC values for all subgroups are presented in Figure 4D.

## 4 Discussion

In this study, GP5 and TTCR were identified as significant predictors of OS‐PC. Additionally, ALP (IFCC) > 130 IU/L, LDH (IFCC) > 300 IU/L, and TTCR were independent predictors of OS‐CRPC. These findings align with previous research; Lim et al. [9] found GP5 and ≥ 4 bone metastases to be prognostic for 201 mCSPC patients, while a Japanese cohort of 1092 mCSPC patients also identified GP5 as a predictor for PC‐specific survival [10]. In our previous study, we identified three prognostic factors for patients with mCSPC—GP5, bone scan index ≥ 1.5, and LDH (IFCC) ≥ 300 IU/L—and proposed a novel risk stratification system, the CSPC classification developed at Kanazawa University (Kanazawa) [11, 12]. Collectively, these findings support the inclusion of GP5 as a prognostic factor in patients with PC.

ALP, crucial for processes like bone formation and liver function, is widely distributed and reflects bone turnover during early cancer metastasis [13, 14]. LDH, a key metabolic enzyme, regulates tumor nutrient exchange and is released into the bloodstream following tissue injury [15]. Consistent with our findings, both ALP and LDH are established prognostic predictors in prostate cancer patients [11, 12, 13, 16].

Various mechanisms of castration resistance have been reported, including AR overexpression, splice variants, missense mutations, involvement of the glucocorticoid receptor, and alterations in coregulator and transcription factors. Nevertheless, AR signaling remains a central therapeutic target of CRPC treatment [17]. Therefore, patients with a longer TTCR are generally expected to have a longer OS. Multiple retrospective studies have examined the prognostic value of time to castration resistance (TTCR) in metastatic castration‐sensitive prostate cancer (mCSPC) patients refractory to initial ADT. Miyake et al. [5] (*n* = 437) reported that longer TTCR correlated with significantly longer OS‐PC, though not OS‐CRPC. In contrast, Okamoto et al. [18] (*n* = 283) found that TTCR ≥ 12 months was associated with significantly longer OS‐PC and OS‐CRPC. Similarly, Kato et al. [19] (*n* = 158) observed that shorter TTCR predicted significantly shorter OS‐PC. These consistent findings regarding the association between TTCR and OS‐PC align with our results. However, the relationship between TTCR and OS‐CRPC remains inconsistent and unclear. It is well established that treatment with ARSIs after progression to mCRPC improves OS‐CRPC [6, 7]. In previous studies, the proportion of patients who received ARSIs after progression to mCRPC was either not reported or inconsistent, which may have contributed to the inconsistent findings regarding the relationship between TTCR and OS‐CRPC. Given that ARSI use after mCRPC progression may have influenced our results, we stratified patients based on ARSI treatment and analyzed OS according to TTCR within each group (Figure S2). The results showed that patients with a longer TTCR had a significantly longer OS‐CRPC regardless of ARSI use. These findings indicated that OS‐CRPC is not necessarily constant across TTCR groups and that prolonging TTCR through early ARSI treatment may not be an absolute requirement for improving survival after mCRPC progression.

No previous studies have specifically investigated the association between TTCR and OS‐CRPC in patients treated with upfront ARSI. It has been indicated that the biological state at the time of CRPC progression may differ between patients treated with conventional ADT and those treated with upfront ARSI [20]. In a cohort study of 829 mCSPC patients, those who progressed to mCRPC after upfront ARSI therapy exhibited more advanced disease, a higher incidence of visceral metastases, and a shorter OS‐CRPC compared to those initially treated with ADT [21]. Therefore, our findings may not be directly applicable to patients receiving upfront ARSI and should be interpreted with caution.

The efficacy of ARSI treatment for mCRPC following ADT is well established [6, 7]. Our study showed that patients receiving ARSI after mCRPC progression had significantly longer OS‐PC and OS‐CRPC in Figures 3 and 4. Notably, those with TTCR ≥ 12 months achieved excellent outcomes with post‐mCRPC ARSI (4‐year OS 91.7%, 5‐year OS 83.0%), surpassing upfront ARSI results in major international phase III trials (ARCHES 71%, TITAN 65.1%, ENZAMET 67%) [22, 23, 24]. These findings suggest that ARSI treatment after progression to mCRPC may be highly effective in patients with TTCR ≥ 12 months. However, identifying patients likely to achieve TTCR ≥ 12 months remains a clinical challenge. We previously reported that in low‐risk mCSPC patients treated initially with ADT, a ≥ 95% decline in PSA at 12 weeks predicted a longer TTCR [15]. Our findings indicated that patients with less than 95% PSA decline at 12 weeks may benefit from early switching to ARSI, which could help identify those who genuinely require upfront ARSI without compromising long‐term outcomes [11].

Asian individuals exhibit higher sensitivity to ADT than White individuals. In a retrospective study involving 165 patients with PC who received ADT in Honolulu, Japanese American men demonstrated significantly better overall and cause‐specific survival compared with White men [25]. Furthermore, multivariate analysis identified race as an independent prognostic factor [25]. The frequency of *HSD3B1* gene mutations—associated with resistance to ADT—is significantly lower in Asian populations (8.7%–17.5%) compared to the United States population (47.9%–52.9%) [26, 27, 28, 29]. These findings indicate that race‐specific differences in response to ADT may play a role in guiding therapy selection for Asian patients.

In a subgroup analysis of the TITAN trial, patients in the ARSI treatment group experienced a significantly higher incidence of radiographic progression without PSA progression compared to those in the placebo group [30]. This type of progression was associated with shorter radiographic progression‐free survival [30]. Furthermore, we previously reported that ARSI treatment is a risk factor for developing new visceral metastases [2]. These findings indicated that selectively identifying patients who do not require upfront ARSI may help avoid these potential disadvantages.

In the patient group with TTCR < 12 months, ARSI treatment after progression to mCRPC significantly prolonged OS‐CRPC compared with those who did not receive ARSI. However, there was no significant difference in OS‐PC. Initiating treatment with ADT or CAB alone in such patients may result in suboptimal outcomes. Therefore, a more intensive treatment approach—such as a triplet regimen combining ADT, DTX, and an ARSI—may be more appropriate.

This study has several limitations. All patients were of Japanese ethnicity, and the analysis was retrospective. Moreover, treatment decisions were made at the discretion of the attending physicians, which may have introduced bias. This cohort includes patients with mCSPC who received treatment over a span of approximately 20 years, during which historical advancements in treatment strategies for CRPC may have influenced OS. Although our findings are clinically meaningful, the limited statistical robustness due to the small sample size and retrospective nature of the study warrants cautious interpretation. Future studies with larger cohorts are needed to validate these results.

In conclusion, in this cohort of mCSPC patients who initiated treatment with CAB, a shorter TTCR and the presence of Gleason pattern 5 were associated with poorer OS‐PC. Additionally, elevated ALP (IFCC) > 130 IU/L, LDH > 300 IU/L (IFCC), and a short TTCR were predictive of worse OS‐CRPC. A shorter TTCR was linked to unfavorable outcomes in both OS‐PC and OS‐CRPC. In contrast, patients with TTCR ≥ 12 months showed significantly better survival even when treated with ARSIs after disease progression to mCRPC. These findings may help reconsider the role of CAB in Asians, including Japanese.

## Author Contributions

**Hiroaki Iwamoto:** conceptualization, investigation, methodology, software, data curation, validation, formal analysis, writing – original draft, writing – review and editing, visualization, project administration, resources, supervision. **Tomohiro Hori:** data curation, investigation. **Takahiro Inaba:** data curation, investigation. **Ryunosuke Nakagawa:** investigation, data curation. **Taiki Kamijima:** investigation, data curation. **Hiroshi Kano:** investigation, data curation. **Tomoyuki Makino:** investigation, data curation. **Renato Naito:** investigation, data curation. **Hiroshi Yaegashi:** investigation, data curation. **Takahiro Nohara:** investigation, data curation. **Kazuyoshi Shigehara:** investigation, data curation. **Kouji Izumi:** supervision, data curation, investigation. **Atsushi Mizokami:** supervision, data curation, investigation.

## Consent

Consent for the use of patient medical record data in this study was obtained using an opt‐out approach.

## Conflicts of Interest

The authors declare no conflicts of interest.

## Supporting information


**Figure S1.** Kaplan–Meier survival curves for OS‐PC and OS‐CRPC stratified by CHAARTED. (A) OS‐PC according to CHAARTED criteria. (B) OS‐CRPC according to CHAARTED criteria. (C) OS‐PC according to TTCR in patients with CHAARTED high volume. (D) OS‐CRPC according to TTCR in patients with CHAARTED high volume. (E) Median OS‐PC for each group. (F) Median OS‐CRPC for each group.
**Figure S2.** Kaplan–Meier survival curves for OS‐PC and OS‐CRPC stratified by TTCR. (A) OS‐PC according to TTCR in patients who received ARSI after mCRPC progression. (B) OS‐PC according to TTCR in patients treated without ARSI after mCRPC progression. (C) OS‐CRPC according to TTCR in patients treated with ARSI after mCRPC progression. (D) OS‐CRPC according to TTCR in patients treated without ARSI after mCRPC progression. (E) Median OS‐PC for each group. (F) Median OS‐CRPC for each group.
**Table S1.** Patient characteristics at the time of mCRPC progression.
**Table S2.** Breakdown of visceral metastases.

## Data Availability

The data presented in this study are available in the article and Supporting Information.
