# Supplementary material for: Prognostic Impact of Time to Castration Resistance on Overall Survival in Patients With Metastatic Castration‐Sensitive Prostate Cancer
Source: Int J Urol. 2025 Aug 19;32(11):1640–9. doi: 10.1111/iju.70197 (PMC12586795; doi:10.1111/iju.70197)
Supplement: Supplementary file 1 — Figure S1. Kaplan–Meier survival curves for OS‐PC and OS‐CRPC stratified by CHAARTED. (A) OS‐PC according to CHAARTED criteria. (B) OS‐CRPC according to CHAARTED criteria. (C) OS‐PC according to TTCR in patients with CHAARTED high volume. (D) OS‐CRPC according to TTCR in patients with CHAARTED high volume. (E) Median OS‐PC for each group. (F) Median OS‐CRPC for each group. Figure S2. Kaplan–Meier survival curves for OS‐PC and OS‐CRPC stratified by TTCR. (A) OS‐PC according to TTCR in patients who received ARSI after mCRPC progression. (B) OS‐PC according to TTCR in patients treated without ARSI after mCRPC progression. (C) OS‐CRPC according to TTCR in patients treated with ARSI after mCRPC progression. (D) OS‐CRPC according to TTCR in patients treated without ARSI after mCRPC progression. (E) Median OS‐PC for each group. (F) Median OS‐CRPC for each group. Table S1. Patient characteristics at the time of mCRPC progression. Table S2. Breakdown of visceral metastases. [file IJU-32-1640-s001.docx]

**Table S1.** Patient characteristics at the time of mCRPC progression

| At the time of mCRPC progression |  | Time to castration resistance, months | | | |  |
| --- | --- | --- | --- | --- | --- | --- |
| Characteristics | All | ≤6 | 6< TTCR ≤12 | 12< TTCR ≤18 | >18 | *p-value* |
| Patients, n | 160 | 22 | 58 | 32 | 48 |  |
| Median age, year | 72.5 | 72 | 73 | 73 | 74 | 0.28 |
| Median PSA,ng/mL | 5.2 | 29.7 | 5.8 | 6.5 | 1.5 | 0.002 |
| Median Hb, g/dL | 12. | 12.4 | 12.4 | 12.3 | 12.7 | 0.02 |
| Median LDH (IFCC), IU/L | 206 | 260 | 201 | 233 | 206 | 0.31 |
| Median ALP (IFCC), IU/L | 92 | 13 | 101 | 98 | 72 | 0.003 |
| Visceral metastasis |  |  |  |  |  | 0.12 |
| No | 107 (66.9) | 14 | 38 | 23 | 32 |  |
| Yes | 23 (14.4) | 7 | 9 | 3 | 4 |  |
| Unknown | 30 (18.8) | 1 | 11 | 6 | 12 |  |
| Treatment after CRPC progression, n (%) |  |  |  |  |  |  |
| ARSI (+) | 93 (58.1) | 11 (50.0) | 33 (56.9) | 19 (59.4) | 30 (62.5) | 0.76 |
| Chemotherapy (+) | 67 (41.9) | 11 (50.0) | 25 (43.1) | 13 (40.6) | 18 (37.5) | 0.26 |

mCRPC: metastatic castration-resistant prostate cancer; PC: prostate cancer; PSA: prostate-specific antigen; Hb: hemoglobin; LDH: lactate dehydrogenase; ALP: alkaline phosphatase; ARSI: androgen receptor signaling inhibitor; TTCR: time to castration resistance

**Table S2.** Breakdown of visceral metastases

|  |  | Time to castration resistance | | | |  |
| --- | --- | --- | --- | --- | --- | --- |
| Characteristics | ALL | TTCR<6 | 6<TTCR<12 | 12<TTCR<18 | 18<TTCR | *p*-value |
| Patients, n | 160 | 22 | 58 | 32 | 48 |  |
| Visceral metastasis at PC diagnosis |  |  |  |  |  | 0.42 |
| Lung | 16 | 1 | 9 | 3 | 3 |  |
| Liver | 2 | 0 | 0 | 1 | 1 |  |
| Visceral metastasis at mCRPC progression |  |  |  |  |  | 0.52 |
| Lung | 15^＊^ | 4^＊^ | 7 | 2 | 2 |  |
| Liver | 7^＊^ | 4^＊^ | 1 | 1 | 1 |  |
| Adrenal gland | 2 | 0 | 1 | 0 | 1 |  |

PC: prostate cancer; mCRPC: metastatic castration-resistant prostate cancer; TTCR: time to castration resistance

^＊^One patient had both lung and liver metastases.


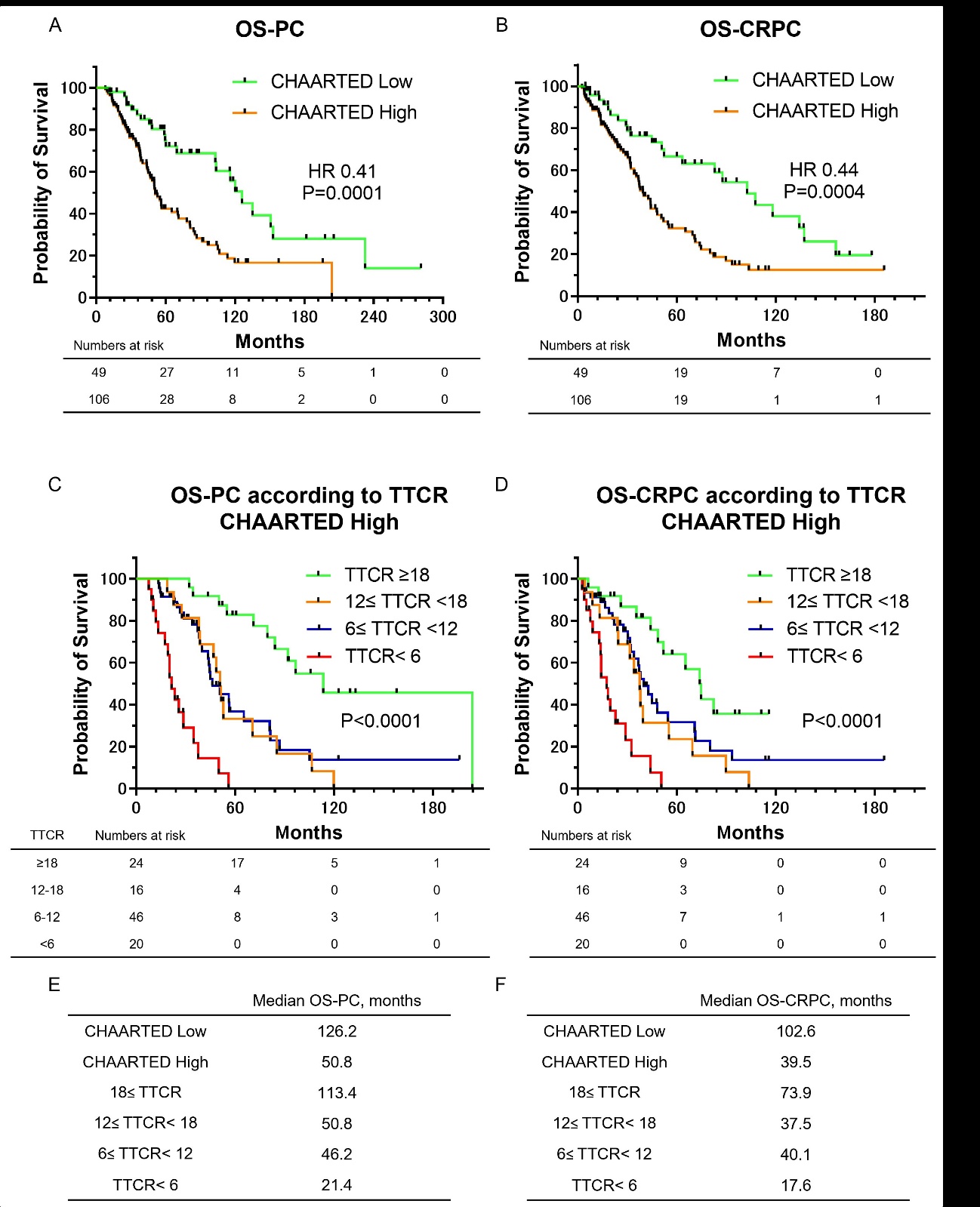
**Figure S1**


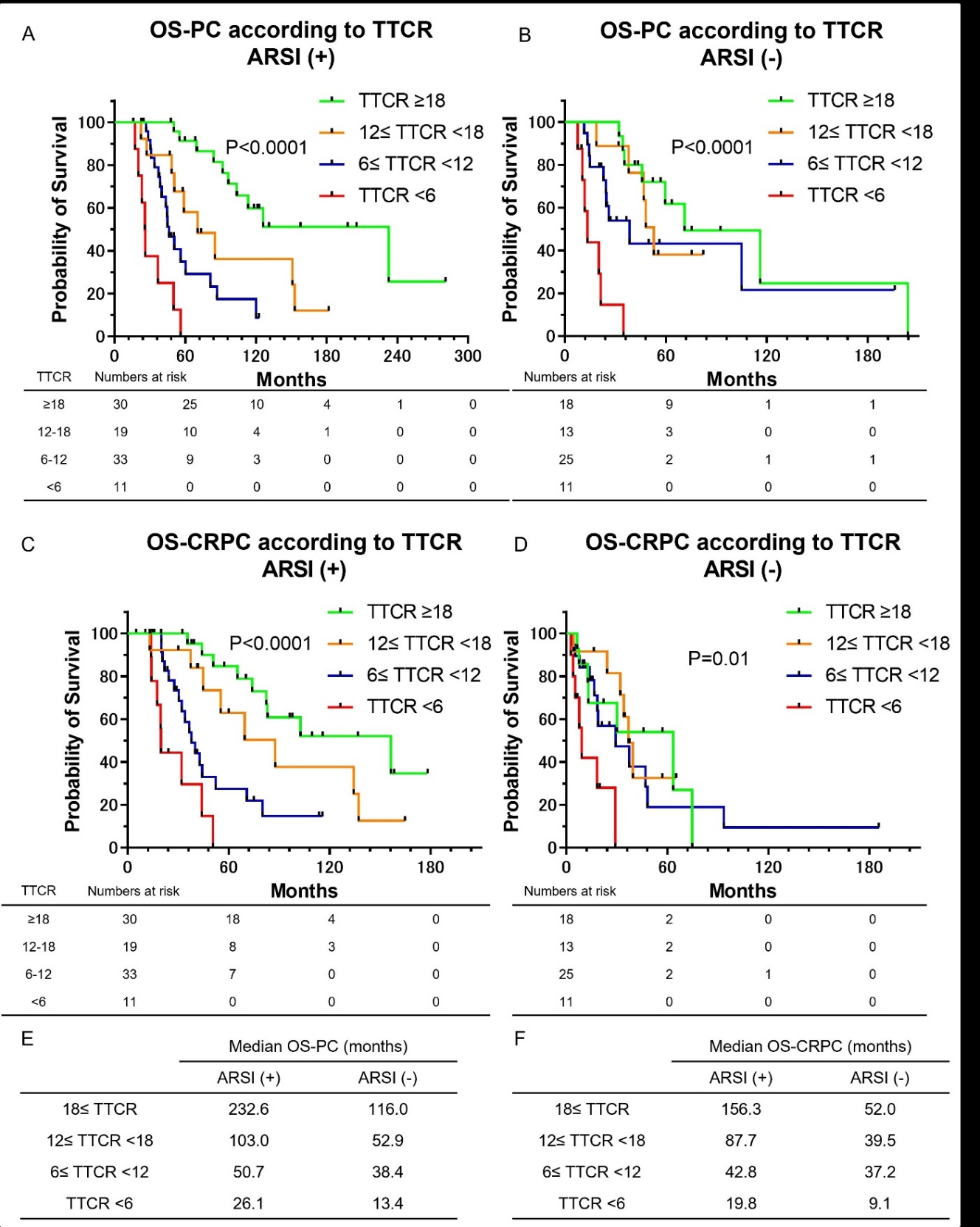
**Figure S2**
